# Overexpression of NREP Promotes Migration and Invasion in Gastric Cancer Through Facilitating Epithelial-Mesenchymal Transition

**DOI:** 10.3389/fcell.2021.746194

**Published:** 2021-10-20

**Authors:** Yuan-jie Liu, Shu-hong Zeng, Yi-dou Hu, Yong-hua Zhang, Jie-pin Li

**Affiliations:** ^1^Department of Oncology, Zhangjiagang TCM Hospital Affiliated to Nanjing University of Chinese Medicine, Zhangjiagang, Jiangsu, China; ^2^Affiliated Hospital of Nanjing University of Chinese Medicine, Jiangsu Province Hospital of Chinese Medicine, Nanjing, Jiangsu, China; ^3^No. 1 Clinical Medical College, Nanjing University of Chinese Medicine, Nanjing, Jiangsu, China

**Keywords:** NREP, gastric cancer, bioinformatics, epithelial–mesenchymal transition, cancer-associated fibroblasts, M2 macrophages

## Abstract

The identification of biomarkers and effective therapeutic targets for gastric cancer (GC), the most common cause of cancer-related deaths around the world, is currently a major focus area in research. Here, we examined the utility of Neuronal Regeneration Related Protein (*NREP*) as a prognostic biomarker and therapeutic target for GC. We assessed the clinical relevance, function, and molecular role of *NREP* in GC using bioinformatics analysis and experimental validation. Our results showed that in GC, *NREP* overexpression was significantly associated with a poor prognosis. Our findings also suggested that *NREP* may be involved in the activation of cancer-associated fibroblasts and the epithelial–mesenchymal transition (EMT), with transforming growth factor β1 mediating both processes. In addition, *NREP* expression showed a positive correlation with the abundance of M2 macrophages, which are potent immunosuppressors. Together, these results indicate that *NREP* is overexpressed in GC and affects GC prognosis. Thus, *NREP* could be a prognostic biomarker and therapeutic target for GC.

## Graphical Abstract

**(A)** Study Flow chart, **(B)** Diagram depicting the regulation mechanism of *NREP* in the tumorigenesis of gastric cancer.

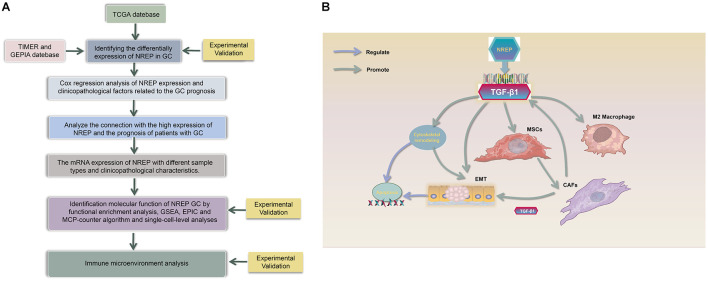

## Introduction

Gastric cancer (GC) is the third most prevalent cancer globally (Son et al., 2021). Both perioperative chemotherapy and preoperative chemoradiotherapy are recommended for the treatment of resectable GC around the world. Of these strategies, perioperative chemotherapy is used most frequently (Al-Batran et al., 2020). The period between the decision to perform surgery and the completion of surgical treatment is called the perioperative period. This period includes the pre-operative, intra-operative, and post-operative stages. Radical surgery is currently the primary curative treatment for resectable GC. In contrast, adjuvant chemoradiotherapy is the standard treatment for unresectable and metastatic GC (De Steur et al., 2021). While chemotherapy improves patient survival, the response to treatment can be variable and unpredictable, with many patients experiencing recurrence and distant metastasis. In advanced GC, the 5-year overall survival (OS) continues to be low at 20–30% (Smyth et al., 2020).

In this era of individualized and precision medicine, molecular targeted therapies and immunotherapies are developing very rapidly, and they have shown great promise in the treatment of GC (Liu and Meltzer, 2017; Pauli et al., 2017). Although previous research has largely focused on targeting malignant cancer cells, an increasing number of studies are now focusing on the tumor microenvironment (TME; Wu and Dai, 2017), which includes all non-malignant host cells and non-cellular components, including immune, blood, and endothelial cells; extracellular matrix (ECM); fibroblasts; and mesenchymal stromal cells (MSCs; Rojas et al., 2020).

There has been recent progress in molecular targeted therapies for GC. Trastuzumab in combination with chemotherapy has been found to improve survival in human epidermal growth factor receptor 2-positive GC (Meric-Bernstam et al., 2019). However, few anti-vascular molecular targeted agents have been identified for advanced GC. Although ramucirumab and apatinib have been approved for second- and third-line treatment, a drug for first-line treatment is still unavailable (Wilke et al., 2014; Scott, 2018). This is likely because of the complex TME of GC and the lack of accurate predictive biomarkers. Therefore, identifying specific biomarkers, targeting the tumorigenic stroma, and reducing the number of immunosuppressive macrophages may be helpful in GC treatment and may also hold the key to improving survival in this cancer.

Neuronal Regeneration Related Protein, which binds to the transforming growth factor β1 (TGF-β1) latency-related protein, is an intracellular polypeptide (8 kDa, 68 amino acids long) that is highly conserved across species and is expressed in the brain, smooth muscles, regenerated tissue, and malignant glioblastomas (Stradiot et al., 2018). *NREP* regulates the expression of TGF-β1 not only at the translational but also at the transcriptional level (Li et al., 2016). *NREP* also regulates myofibroblast differentiation and fibrosis and promotes embryo development, wound healing, and nerve and lung regeneration (Studler et al., 1993; Duan et al., 2019). The molecular physiology of wound healing is thought to be very similar to that of cancer progression (Margadant and Sonnenberg, 2010). During wound healing, epithelial-mesenchymal transition (EMT) confers motility and invasiveness to epithelial cells, thereby allowing them to travel to the wound site and repair tissue damage. Similarly, once cancer cells enter the EMT phase, they become locally invasive, and this is the first step in tumor metastasis. Many important signaling pathways and molecules involved in wound healing also regulate tumor cell proliferation and metastasis (Derynck and Weinberg, 2019). Given these findings and the results of enrichment analysis for *NREP*-related genes, we hypothesized that NREP may have a powerful role in promoting EMT in tumors. Further, we speculated that TGF-β1 could be an important mediator in the effects of NREP. TGF-β1 is a well-known key factor in the TME and can promote the reprogramming of tumor-infiltrating cells, including tumor-associated macrophages (TAMs) and tumor-associated fibroblasts, enabling them to play a decisive role in tumor survival and progression. Signaling crosstalk between cancer cells and mesenchymal cells ultimately leads to an environment that supports tumor growth and metastasis. Recently, high *NREP* expression was observed around the rims of invasive human glioma tumors. Furthermore, *NREP* knockdown in human glioma cells (SF767) has been found to reduce their migration ability *in vitro* (Mariani et al., 2001; Yao et al., 2015, 2017). However, data on the role played by *NREP* in tumorigenesis, particularly in GC, are limited. Therefore, we examined the mechanistic role of *NREP* in GC and its development. We also assessed its prognostic value in GC along with its potential as a target for cancer therapy.

## Materials and Methods

### Antibodies and Reagents

A complete list of reagents and antibodies is provided in Supplementary Material 1. All the concentrations were chosen based on previous studies or manufacturer’s instructions. The detailed screening protocol is also presented in Supplementary Material 1.

### Cell Culture

AGS (moderately differentiated GC cells), HGC27 (undifferentiated GC cells), GES-1 (healthy gastric epithelial cells), and THP-1 cells (human monocytic cells) were purchased from the cell bank of the Chinese Academy of Sciences (Shanghai, China). MKN74 and MKN45 cells (well and poorly differentiated GC cells, respectively) were purchased from the Japanese Collection of Research Bioresources Cell Bank. Human MSCs were purchased from Cyagen Biosciences (Guangzhou, China). GC and THP-1 cells were cultured in RPMI-1640 medium with 10% fetal bovine serum (FBS), and GES-1 cells and human MSCs were cultured in DMEM with 10% FBS. All cells were incubated at 37°C in 5% CO_2_.

### RNA Extraction and Real-Time Quantitative PCR

Quantitative RT-PCR was performed using the protocol provided in previous studies (Bustin et al., 2005). Total RNA was extracted from cells using the TRIzol reagent. cDNA was synthesized via reverse transcription using the manufacturer’s protocol. β-actin was chosen as the internal control. The primers were as follows: β*-actin* (F): 5′-GCGTGACATTAAGGAGAAGC-3′; β*-actin* (R): 5′-ACGTCACACTTCATGATGG-3′; *NREP* (F):5′ TTGAGCGAATGCTACCAGAG-3′; and *NREP* (R):5′-AGGCG AGGCTACGGAAAG -3′.

### Western Blot Assessment

The protocol for western blotting was based on previous studies (Hnasko and Hnasko, 2015). Target/β-actin bands were identified with a gel image processing system (ChemiDoc XRS+). Subsequently, relative protein levels were calculated.

### Ethics Statement and Specimen Collection

The study’s protocol was approved by the ethics committee of the Jiangsu Province Hospital of Chinese Medicine, and informed consent was obtained from clinicians and patients (2019NL-166-02). GC tissue and the surrounding non-tumorous tissue (margin, 5 cm) were collected during surgery from 30 previously treatment-naïve patients with GC at the Jiangsu Provincial Hospital of Traditional Chinese Medicine. Tumors were staged and graded using the 8th edition of the American Joint Committee on Cancer tumor-node-metastasis staging system (Ji et al., 2018). After extraction, tissue specimens were washed with cold phosphate-buffered saline and immediately placed in liquid nitrogen. They were then transferred and stored at −80°C until further examination.

### Immunohistochemistry

The protocol used for Immunohistochemistry (IHC) was based on earlier studies (Nizioł et al., 2021). Images were obtained using a NIKON Eclipse Ni-E microscope (NIKON, Japan; original magnification, ×400). The H-SCORE (range 0–300, higher scores indicating stronger positive staining) was calculated as described previously (Yang et al., 2019).

### Lentiviral Vector Construction and Transfection

We used lentiviral vectors for overexpressing and knocking down *NREP.* Viruses were designed, synthesized, and produced by GeneChem Corporation. Transfection was performed according to the supplier’s protocol. HGC27 and MKN74 cells were transduced with the recombinant lentiviruses using 2 μg/mL polybrene for 24 h. Subsequently, we identified stably transfected GFP-expressing cells using 1.5 μg/mL puromycin. We assessed *NREP* overexpression and knockdown as well as transduction efficiency using western blots.

### Enzyme-Linked Immunosorbent Assay

We examined cell supernatants for TGF-β1 levels using the TGF-β1 Enzyme-linked immunosorbent assay (ELISA) Kit based on the given instruction manual. A microplate reader (BioTek Synergy HT) was used to examine optical density at 450 nm.

### Colony Formation Assays

We assessed the clonogenic ability of cells using a clone formation assay, as described previously (Li et al., 2012). The number of colonies was counted using a compound light microscope (Olympus BX53, Japan).

### Xenograft Tumor Model

All animal experiments were approved by the ethics committee of the Jiangsu Province Hospital of Chinese Medicine (2021-5-062). Twenty-four 4-week-old male BALB/c nude mice were obtained from the Beijing Institute of Biomedicine (Beijing, China; Certificate No. SYXK2019-0010). MKN74 cells transfected with sh-NREP, oe-NREP, and NC and control cells (4 × 10^6^ cell/mouse) were injected subcutaneously into the right armpit region of the mice (*n* = 6 per group). 7 days later, tumor formation was observed beneath the skin. The maximum (a) and minimum tumor diameter (b) were measured twice weekly. On day 15, the mice were euthanized and all tumors were collected. Tumor volume was calculated (V = 1/2ab^2^), and the growth curves of the subcutaneous xenografts were drawn.

### Wound Healing Assay

The protocol used for the wound healing assay was based on earlier studies (Pasquale et al., 2020). Cell migration toward the scratch zone was photographed using an inverted fluorescence microscope (Olympus CKX-41, Japan; ×200 magnification) every 12 h.

### Transwell Migration Assay

Cell invasion was assessed using a transwell assay based on a previously published protocol (Misra et al., 2021). The membrane in the chamber was cut and imaged using light microscopy (Olympus BX53, Japan; ×200 magnification), and cell counts were obtained using Image J software.

### TUNEL Staining

The TUNEL apoptosis detection kit was used to perform the TUNEL assay, as described previously (Telegina et al., 2019). TUNEL-negative (blue) and TUNEL-positive (red) cells were observed using a fluorescence microscope (Olympus CKX-41, Japan; ×200 magnification).

### Immunofluorescence Staining

The protocol used for immunofluorescence staining was based on earlier studies (Donaldson, 2015). Immunofluorescence staining was observed using an epi-fluorescence microscope (Olympus, BX60-32FB2-A03) and different filters and imaged using an Olympus, DP50 camera (×400 magnification).

### Establishment of a Co-culture Unit

A non-contact co-culture unit of MSCs and GC cells was established using a co-culture transwell system (upper chamber, GC cells; lower chamber, MSCs; Long et al., 2019). The culture medium was changed every 48 h. After 4 days of non-contact co-culture, the culture in the lower chamber was terminated and cells were harvested for other experiments.

THP-1 cells (1 × 10^5^ cells/mL) were treated with phorbol 12-myristate 13-acetate (PMA; 10 ng/mL) for 48 h to allow the induction of macrophage differentiation (Genin et al., 2015). PMA-containing medium was replaced with serum-free medium, and the cells were cultured for 24 h. 2 days before the co-culture experiment, cells (1 × 10^5^ cells/mL) from the control, knock-down (sh-NREP), overexpression (oe-NREP), and negative control (NC) groups were seeded onto 0.4-μM transwell inserts. For co-culture, the culture medium in the inserts with GC cells was removed and transferred to the top of the pates with differentiated THP-1 cells. After 48 h of further co-culturing, cells were obtained, and immunofluorescence staining was performed.

### Statistical Analysis

Data were reported as the mean ± standard deviation. We used *t*-tests and one-way ANOVA to perform comparisons between two groups and among multiple groups, respectively. All data were analyzed using SPSS 26.0 (SPSS Inc., United States) and illustrated using GraphPad Prism 8.0 (GraphPad Software, Inc., United States). All experiments were carried out at least thrice. ^∗∗^*P* < 0.01 and ^∗^*P* < 0.05 were considered statistically significant.

### Expression Analysis

The expression of *NREP* in GC was first investigated using the TIMER and^1^ and GEPIA databases^2^ (Pan et al., 2019; Yuan et al., 2019). The Cancer Genome Atlas (TCGA)-Stomach Adenocarcinoma (STAD) cohort and Fei et al. (2018), Li et al (2020), and Shan et al. (2021) datasets were then used to further confirm the differential expression of *NREP* (Rossari et al., 2018; Tian et al., 2018). The Human Protein Atlas database^3^, which contains data on >11,200 unique proteins, is the biggest, most comprehensive database on protein distribution in human tissues and cells (Thul and Lindskog, 2018).

### Cox Model Establishment and Clinical Value Analysis of NREP in Gastric Cancer

Multivariate Cox regression analysis of TCGA-STAD data was used to identify whether *NREP* could be an independent prognosticator for GC. *P* values, hazard ratios (HRs), and 95% confidence intervals (CIs) were obtained using the “forest plot” R package.

The differences in *NREP* levels were analyzed based on various classification parameters, such as the T/N/M stage, pathologic stage, and histologic grade.

Differences in survival based on *NREP* expression were examined using Kaplan–Meier survival analysis and log-rank tests. *P* values, HRs, and 95% CIs were obtained using log-rank tests and univariate Cox proportional hazards regression. Time receiver operating characteristic (ROC) analysis was applied to calculate the accuracy of prognostication based on *NREP* levels.

### Gene Enrichment Analysis

The GeneMANIA database was used to identify genes that showed *NREP*-lined expression and to explore their potential functions (Franz et al., 2018). Genes co-expressed with *NREP* were identified based on TCGA-STAD data (criteria: |logFC > 3| and *P* < |0.05|). Subsequently, we conducted functional enrichment analysis of *NREP* and the identified genes using the Enrichr database (Kuleshov et al., 2016).

To analyze the connections among proteins, genes co-expressed with *NREP* were assessed using the Search Tool for the Retrieval of Interacting Genes database (STRING)^4^ (Szklarczyk et al., 2021); we visualized the results obtained after setting the minimum interaction score to 0.4 in Cytoscape (Doncheva et al., 2019). In addition, based on the constructed protein–protein interaction network (PPI), important modules were screened out using the Molecular Complex Detection (MCODE) tool. Hub genes were obtained by setting the following cutoffs: degree cutoff value = 2, node density cutoff value = 0.1, node score cutoff value = 0.2, k-core = 2, and maximum depth = 100. Finally, the relationship between the levels of these hub genes and GC prognosis was examined using TCGA-STAD data.

Gene set enrichment analysis (GSEA) was performed using the Broad Institute GSEA software 3.0. The geneset “subset of GO (Gene Ontology)” (Molecular Signatures Databases; http://www.gsea-msigdb.org/gsea/msigdb/index.jsp) was used for GO enrichment analysis (Powers et al., 2018). Statistical significance was defined at a normal *P* value < 0.05. The GSCALite online tool was used to explore the relationship between the 10 hub genes and EMT, and we calculated the co-expression relationship between NREP and 6 classical factors of EMT using TCGA-STAD data (Liu et al., 2018). In addition, single-cell analysis was conducted based on GSE134520 to find more evidence on the potential functions of NREP.

### Immune Cell and Stromal Cell Analysis

The correlation between *NREP* expression levels and fibroblast levels was first calculated based on the Explicitly Parallel Instruction Code (EPIC) and Mixed Complementarity Problem (MCP)-counter algorithm from the TIMER web tool (Sturm et al., 2019). GC patients were grouped into high- and low-expression cohorts based on the median *NREP* expression, and differential analysis was performed to identify the differentially abundant cells. Furthermore, CIBERSORT — a high-performance computational method used for quantifying cellular components from bulk-tissue gene expression profiles — was used to accurately estimate immune infiltration (Chen et al., 2018). Spearman’s rank correlation coefficients were calculated for pairwise correlation comparisons; *P* < 0.05 was defined as statistically significant. All findings were illustrated using “ggplot2” and “pheatmap.”

## Results

### NREP Expression in Gastric Tumors

**FIGURE 1:**
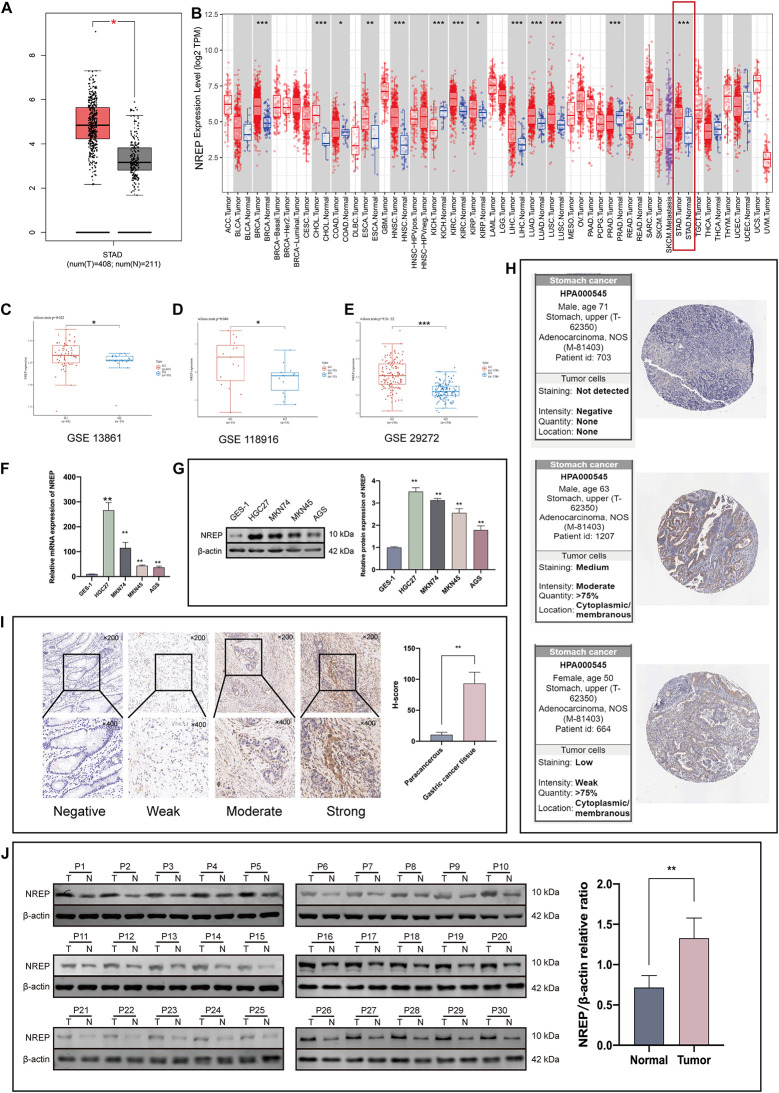
*NREP* levels in gastric cancer (GC) tissue. **(A)** Expression levels of *NREP* in GC based on The Cancer Genome Atlas (TCGA)-STAD data. **(B)** *NREP* mRNA levels in GC and normal tissue based on the TIMER database. **(C–E)** Public datasets from Gene Expression Omnibus (Fei et al., 2018; Li et al, 2020; Shan et al., 2021) used to verify *NREP* mRNA levels in GC. **(F,G)** *NREP* mRNA **(F)** and protein expression **(G)** in normal gastric epithelial cells and GC cells. **(H)** NREP immunohistochemistry in GC tissue based on data from the Human Protein Atlas. **(I)** Intensity of NREP immunohistochemistry staining and NREP expression levels in paracancerous and GC tissue (*n* = 30). **(J)** NREP expression in GC tissues (T) and paired non-tumorous tissue (N) evaluated using western blotting (*n* = 30). (NS: not significant, *P < 0.05, **P < 0.01, and ***P < 0.001).

The Cancer Genome Atlas-Stomach Adenocarcinoma data showed that *NREP* expression was higher in GC tissues than in normal tissues (Figure 1A; *P* < 0.05). Moreover, the analysis of TIMER and TCGA data also showed high *NREP* expression in GC (Figure 1B).

We further explored the expression of *NREP* in GC tissues using data from the Gene Expression Omnibus (GEO) database. Data from the Fei et al. (2018), Li et al (2020), and Shan et al. (2021) datasets indicated a significant difference in NREP expression between GC tissues and adjacent tissues (Figures 1C–E). Western blot, RT-PCR, and IHC staining also revealed that NREP was over-expressed in GC cells and tissue. The mean H-SCOREs for NREP expression in GC and paracancerous tissue were 93.15 ± 18.21 and 10.49 ± 3.94, respectively (Figures 1F–J; *P* < 0.01, ANOVA). NREP protein expression in GC was further verified using IHC data from The Human Protein Atlas. NREP was found to be primarily expressed in the cell membrane and cytoplasm (Figure 1H).

### Prognostic Value of NREP Expression in Gastric Cancer

**FIGURE 2:**
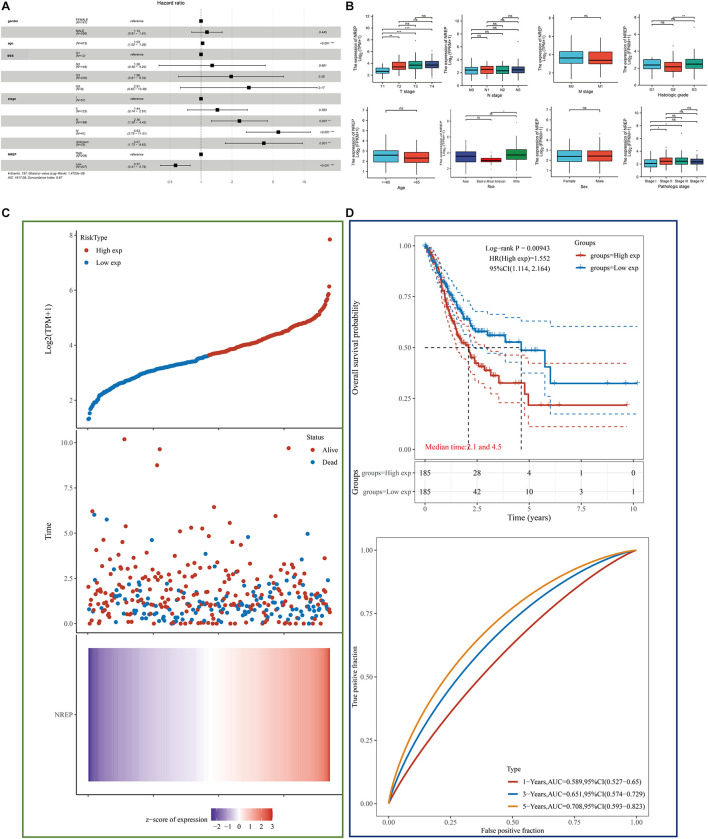
Diagnostic value of *NREP* expression in gastric cancer (GC) based on clinical characteristics. **(A)** Forest plot showing data on Sex, Age, tumor Grade/Stage, and *NREP* expression. **(B)** Association of *NREP* mRNA expression with T/N/M stage, histologic grade, pathologic stage, sex, race, and age in GC patients. **(C)** Patients were divided into low- and high-risk groups according to the median *NREP* expression. From top to bottom: The curve of risk score. Survival status of the patients and more dead patients corresponding to the higher risk score. Heatmap of *NREP* expression. The horizontal coordinates all represent samples, and the samples are ordered consistently. **(D)** Kaplan–Meier survival analysis and time-dependent receiver operating characteristic analysis based on *NREP* levels. (ns: no significance, *P < 0.05, **P < 0.01, ***P < 0.001).

Multivariate analysis of TCGA-STAD data revealed that *NREP* overexpression, age, and tumor stage were related with a poor prognosis in GC (*P* < 0.001; Figure 2A). In particular, *NREP* levels were identified as an independent predictor of survival in GC patients. The relationship of *NREP* levels with the clinicopathological characteristics of GC patients — including T/N/M stage, histologic grade, pathologic stage, gender, and race — is illustrated in Figure 2B. The mRNA levels of *NREP* only showed a correlation with T stage (*P* < 0.01, *P* < 0.001), and no such relationship was observed for other clinical features. Further analysis of the prognostic value of *NREP* using TCGA-STAD data showed that the low-risk group had a longer duration of survival than did the high-risk group (Figure 2C; *P* < 0.05). Additionally, according to TCGA-STAD data, the areas under the ROC curves of *NREP* expression for 1-, 3-, and 5-year OS were 0.589, 0.651, and 0.708, respectively, (Figure 2D). In summary, the results showed that *NREP* overexpression could be used as an indicator for OS in GC.

### Functional Enrichment Analysis of NREP

**FIGURE 3:**
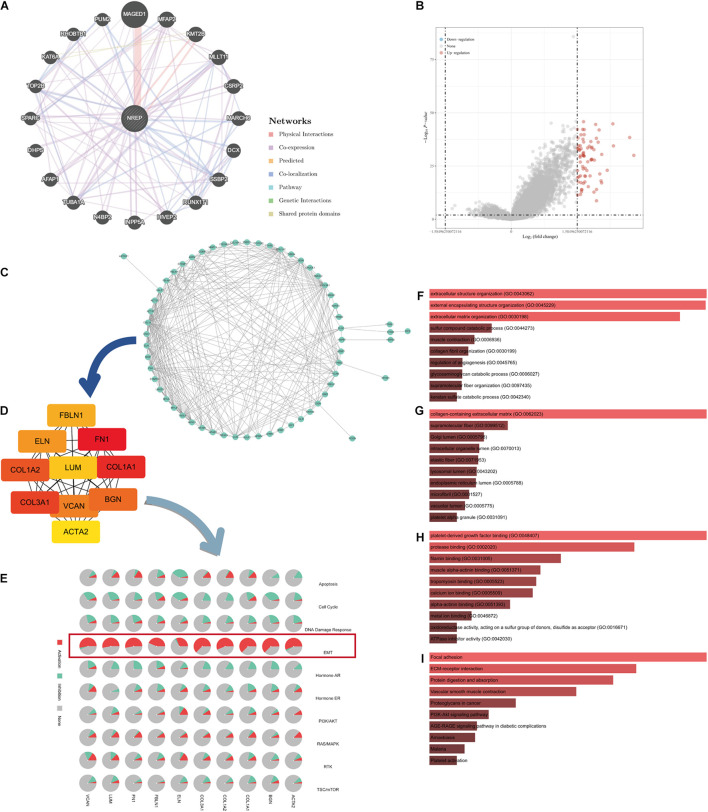
Protein–protein interaction (PPI) network and enrichment analysis. **(A)** GeneMANIA-based PPI; *NREP* is at the core of this network. **(B)** Volcano map of genes showing differential expression after a change in *NREP* levels. Red dots, up-regulated genes; blue dots, down-regulated genes; abscissa, differences in gene expression (log2 fold change); and ordinate, significance of these differences (−log10 padj). **(C)** Network of *NREP* and genes with *NREP-*linked expression (positive). **(D)** Hub gene network of *NREP*. **(E)** Relationship between hub genes and epithelial–mesenchymal transition. **(F–I)** Functional enrichment analysis of *NREP*-related genes. **(F)** Biological Processes (BP), **(G)** Cellular Components (CC), **(H)** Molecular Functions (MF), and **(I)** Kyoto Encyclopedia of Genes and Genomes (KEGG).

The functional network of *NREP* and its neighboring genes obtained using GeneMANIA is displayed in Figure 3A. We identified genes showing expression levels positively or negatively correlated with *NREP* expression using TCGA-STAD data and the “DESeq” R package (Figure 3B). The 73 differentially expressed genes (DEGs) were imported into the DEG PPI (Figure 3C). After applying Cytotype MCODE, we identified 10 hub genes among which 8 were up-regulated and 2 were down-regulated (Figure 3D and Supplementary Figures 1A–J). The GSCALite online tool was used to elucidate the relationship between the 10 hub genes and EMT (Figure 3E). Further enrichment analysis suggested that *NREP* may be associated with “extracellular structure organization,” “external encapsulating structure organization,” “extracellular matrix organization,” “collagen-containing extracellular matrix,” “platelet-derived growth factor binding,” “focal adhesion,” and “ECM-receptor interaction” (Figures 3F–I).

Additional survival analysis revealed that the levels of 5 up-regulated genes were associated with the prognosis of GC patients (Supplementary Figures 1K–T).

### Relationship Between NREP and EMT and Its Underlying Mechanism

**FIGURE 4:**
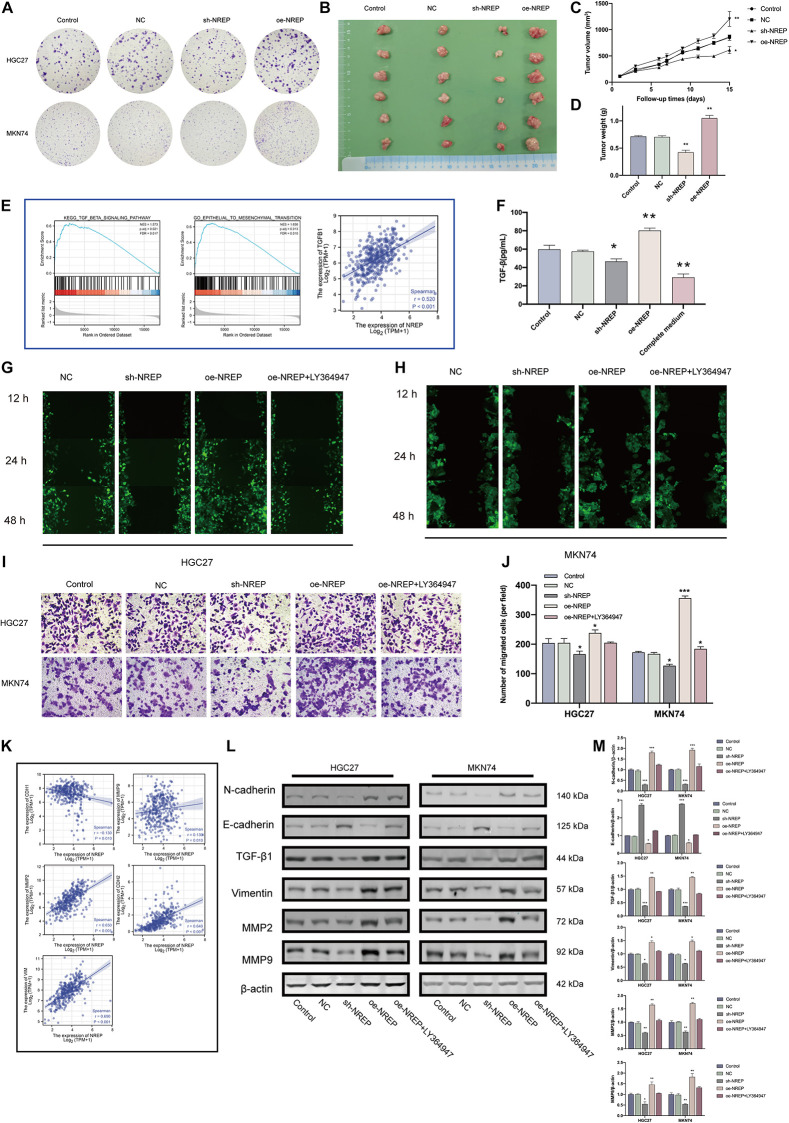
*NREP* overexpression promotes a malignant phenotype in gastric cancer (GC). **(A)** Clone formation capacity of GC cells transfected with the NC, sh- NREP, and oe-NREP constructs assessed using the clone formation assay. **(B)** Xenograft tumors from nude mice. **(C,D)** Tumor volume **(C)** and weights **(D)** of xenografts from nude mice **(E)** Gene Set Enrichment Analysis (GSEA) of *NREP* and correlation analysis between *NREP* and *TGF-*β*1* expression using TIMER. **(F)** TGF-β1 levels in the supernatant of GC cells transfected with NC, sh-NREP, and oe-NREP examined using ELISA. **(G,H)** Migratory ability of different group of GC cells [**(G)**: HGC27, **(H)**: MKN74] examined using wound healing assays. **(I,J)** The invasion ability of GC cells after transfection (**I**; magnification, ×200); the relative invasive cell number is shown toward the right in **(J)**. **(K)** Correlation coefficient circles for NREP and EMT-related genes (TIMER). **(L)** Expression of EMT-related proteins examined using western blots after the transfection of GC cells with NC, sh-NREP, and oe-NREP constructs and treatment with 5 μM LY364947, an inhibitor specific to TGF-β type I receptor. **(M)** Statistical analysis of western blot results. (*P < 0.05, **P < 0.01, and ***P < 0.001).

*In vitro NREP* silencing in HGC27 and MKN74 cells using shRNA constructs significantly down-regulated *NREP* expression (Supplementary Figure 2; *P* < 0.01). *NREP* silencing also decreased tumor cell clone formation (Figure 4A). In contrast, the opposite trend was observed in cells overexpressing *NREP*. Moreover, stable *NREP* overexpression in MKN74 cells promoted the formation of subcutaneous xenograft tumors *in vivo* (Figures 4B–D; *P* < 0.01). GSEA for *NREP* revealed the potential role of *NREP* in “epithelial–mesenchymal transition” and “TGF-beta signaling.” Analyses using TIMER data revealed a positive correlation between NREP and TGF-β1 expression (*R* = 0.520, *P* < 0.001; Figure 4E). Subsequent *in vitro* experiments using ELISA revealed a reduction in TGF-β1 levels in culture medium after *NREP* silencing (*P* < 0.05; Figure 4F), and the opposite trend was observed when *NREP* was overexpressed. *NREP* overexpression was also found to increase EMT-related phenotypes such as the migration and invasion of GC cells and the expression of EMT-associated proteins. However, this effect was attenuated after treatment with the TGF-β signaling kinase inhibitor LY364947 (Figures 4G–J). In addition, cell viability assays showed that the selected concentration of LY364947 did not affect cell proliferation (Supplementary Material 1). Further analyses based on TIMER data also revealed a positive correlation between the expression of *NREP* and that of *CDH2* (*R* = 0.64, *P* < 0.001), *MMP2* (*R* = 0.65, *P* < 0.001), *MMP9* (*R* = 0.13 *P* = 0.01), and *VIM* (*R* = 0.69, *P* < 0.001). Further, *NREP* expression was found to show a negative correlation with *CDH1* expression (*R* = −0.130, *P* = 0.01; Figure 4K). Therefore, *NREP* silencing and overexpression altered the levels of EMT-related proteins (Figures 4L,M).

### Relationship of NREP Expression With Cytoskeletal Remodeling and Gastric Cancer Cell Apoptosis

**FIGURE 5:**
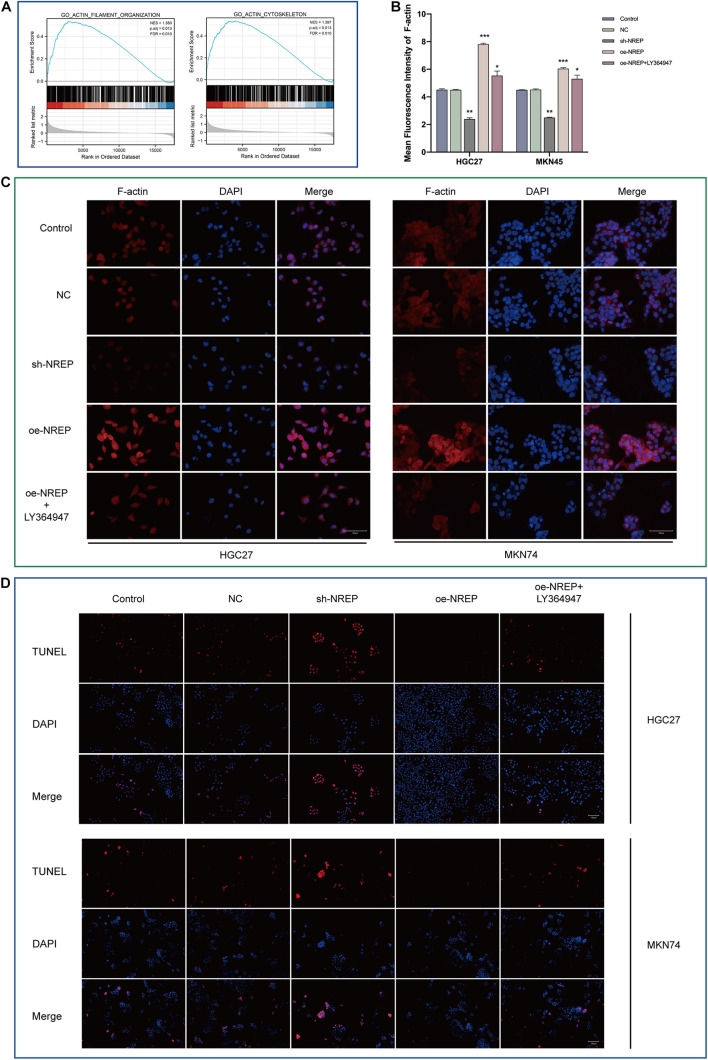
Relationship between NREP and *F*-actin cytoskeleton and cell apoptosis. **(A)** Gene Set Enrichment Analysis (GSEA) of NREP. **(B)** Immunofluorescence intensities. **(C)** *F*-actin levels in gastric cancer (GC) cells (control cells and GC cells transfected with the NC, sh-NREP, and oe-NREP constructs) treated with 5 μM LY364947 detected using immunofluorescence staining (magnification, ×400). **(D)** Apoptosis assessed using a TUNEL assay (TUNEL-positive cells indicated in red; magnification, ×200). (*P < 0.05, **P < 0.01, ***P < 0.001).

Gene set enrichment analysis also revealed functional enrichment for *NREP* under the “ACTIN FILAMENT ORGANIZATION” and “ACTIN_CYTOSKELETON” domains (Figure 5A). Subsequent *in vitro* experiments revealed that *NREP* overexpression caused the up-regulation of F-actin (Figures 5B,C; *P* < 0.01). Moreover, after TUNEL staining, no TUNEL-positive cells were detected in cells overexpressing *NREP*. In contrast, *NREP* knockdown and LY364947 treatment increased the number of TUNEL-positive cells (Figure 5D).

### Relationship of NREP Expression With the Differentiation of Mesenchymal Stromal Cells Into Cancer-Associated Fibroblasts

**FIGURE 6:**
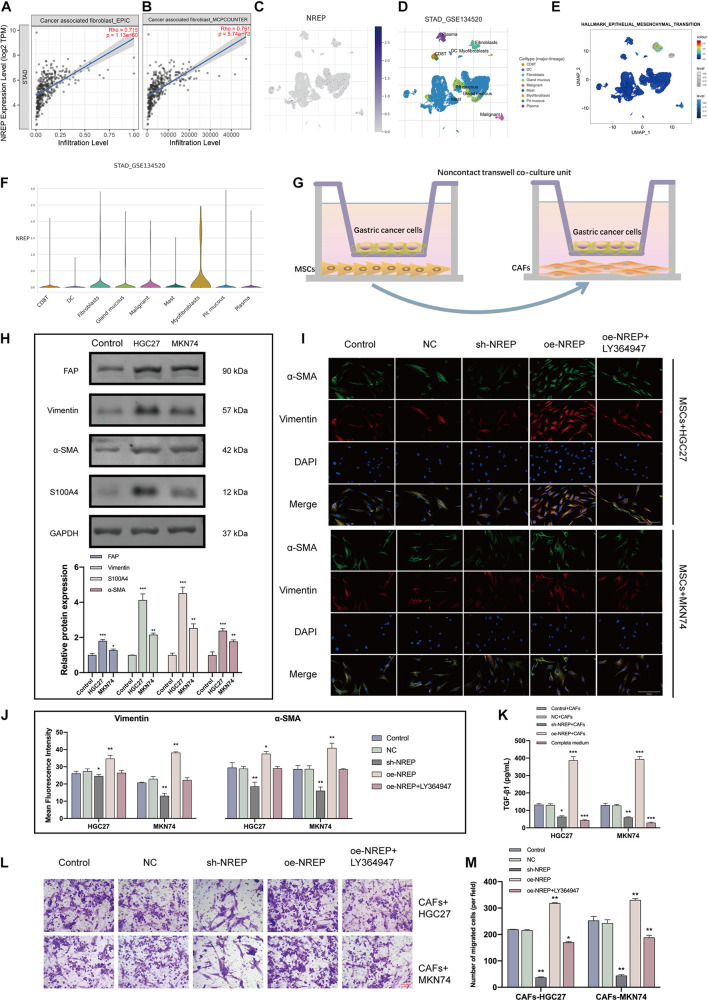
NREP upregulation in gastric cancer (GC) promotes the activation and recruitment of cancer-associated fibroblasts (CAFs). **(A,B)** Correlation of NREP expression with CAFs based on the Fei et al., 2018 **(A)** and Li et al, 2020 **(B)** datasets. **(C)** Uniform manifold approximation and projection (UMAP) plots illustrating the expression of NREP clusters. **(D)** UMAP plots illustrating the GC cell landscape. We found nine cell clusters across 56,440 cells after quality control, dimensionality reduction, and clustering. **(E)** Enrichment score for genes from the Hallmark hypoxia gene set in each cell, obtained using gene set variation analysis. **(F)** Violin plots for GC cell cluster marker genes and *NREP* in different cell types. Expression was measured as log 2 (TP10K + 1). **(G)** A non-contact co-culture unit of MSCs and GC cells established by incubating MSCs with GC cells at a 1:1 ratio. **(H)** After co-culture with GC cells for 4 days, CAF-related markers were examined using western blots. **(I)** Immunofluorescence staining of MSCs co-cultured with GC cells (control cells and GC cells transfected with the NC, sh-NREP, and oe-NREP constructs) treated with 5 μM LY364947 (magnification, ×400). **(K)** After co-culture with different groups of GC cells for 4 days, ELISA assays were used to demonstrate an increase in the TGF-β1 protein levels in the supernatant of the co-culture system. **(L)** The transwell system was used to investigate the ability of GC cells to recruit CAFs. (magnification, ×200). **(M)** The number of migrated cells was counted using Image **(J)**. (*P < 0.05, **P < 0.01, and ***P < 0.001).

The abundance of cancer-associated fibroblasts (CAFs) was found to be a potential prognostic factor in GC (Table 1). Using the EPIC and MCP-counter algorithms and TCGA-STAD data (Figures 6A,B), we found that *NREP* expression was positively correlated with CAF abundance (EPIC: *R* = 0.715, *P* = 1.13e-60 and MCP-counter: *R* = 0.761, *P* = 5.74e-73). Subsequent single-cell-level analyses revealed that *NREP* was mainly expressed in fibroblasts, which are important players in EMT (Figures 6C–F).

**TABLE 1:** The Cox analysis of the Cancer associated fibroblast (CAFs).

	Coef	HR	Se(coef)	95%CI_l	95%CI_u	*p* value
Cancer associated fibroblast_EPIC	1.546	4.692	0.629	1.368	16.093	0.014
Age	0.036	1.036	0.009	1.017	1.055	0
Stage2	0.339	1.404	0.341	0.719	2.74	0.32
Stage3	0.849	2.338	0.318	1.254	4.356	0.007
Stage4	1.567	4.793	0.372	2.31	9.946	0
Gendermale	0.153	1.166	0.191	0.801	1.696	0.423
Purity	−0.422	0.655	0.357	0.326	1.319	0.237

*Coef: regression coefficient; HR: hazard ratio; se (coef): standard error of regression coefficient; CI-L: confidence interval low; and CI-U: confidence interval up.*

Local and recruited MSCs are known to transform into CAFs at close proximity to tumor cells. To test whether *NREP* overexpression in GC facilitates the conversion of MSCs into CAFs, we co-cultured MSCs with GC cells (Figure 6G). After the co-culture of MSCs with GC cells, CAF markers were remarkably up-regulated in MSCs (Figure 6H; *P* < 0.01, *P* < 0.001). Furthermore, we co-cultured MSCs with GC cells showing different levels of *NREP* expression for 4 days. Immunofluorescence staining revealed that α-SMA and Vimentin levels were up-regulated in the *NREP* overexpression group, whereas they were down-regulated in the sh-*NREP* group (Figures 6I,J; *P* < 0.05, *P* < 0.01). Interestingly, ELISA revealed a 4-fold increase in the levels of TGF-β1 in the cell supernatant after MSCs were co-cultured with *NREP*-overexpressing cells (Figure 6K; *P* < 0.05, *P* < 0.05, and *P* < 0.001). Consistent with our previous results, these findings also showed that a TGF-β inhibitor can decrease the ability of MSCs to differentiate into CAFs in a co-culture unit.

Recently, two modes of cancer cell invasion have been defined: collective cancer cell invasion and fibroblast-led collective invasion. Next, we examined the effect of *NREP* overexpression on CAF recruitment through a transwell experiment. We observed that with an increase in *NREP* expression, the ability of GC cells to recruit CAFs was significantly enhanced (Figures 6L,M).

Together, these results indicated that *NREP* may contribute to GC progression by recruiting and activating fibroblasts.

### Relationship of NREP Expression With M2 Macrophage Infiltration

**FIGURE 7:**
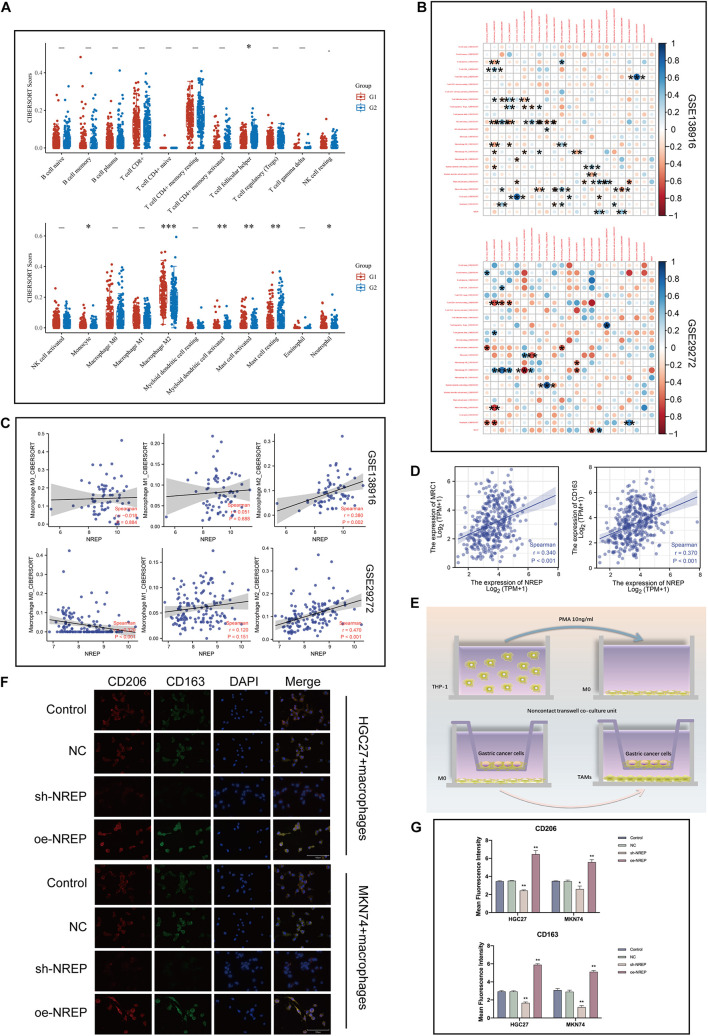
Association of *NREP* expression with the abundance of M2 tumor-associated macrophage infiltration. **(A)** Score distribution of immune cells in gastric cancer (GC) and normal tissues. Horizontal axis, different groups; vertical axis, distribution of gene expression; G1, high *NREP* expression group; and G2, low *NREP* expression group. **(B)** Correlation matrix showing the abundance of 22 types of immune cells. **(C)** Correlation of *NREP* expression levels with macrophage abundance based on Fei et al., 2018 and Li et al, 2020 datasets. **(D)** Correlation of *NREP* expression levels with M2 macrophage markers. **(E)** Schematic diagram for the tumor–macrophage cell co-culture. **(F)** Immunofluorescence staining for CD206 (green) and CD163 (red; magnification, ×400). **(G)** Immunofluorescence expression. (*P < 0.05, **P < 0.01, and ***P < 0.001).

Using CIBERSORT, we found that the proportion of tumor-infiltrating cells was positively correlated with *NREP* levels and the presence of M2 macrophages in patients with GC (*P* < 0.001; Figure 7A). Subsequently, the relationship of *NREP* expression with macrophage polarization was assessed using the Li et al, 2020 and Fei et al., 2018 datasets. The correlation between *NREP* expression and immune cell subpopulations is shown in Figure 7B. Interestingly, *NREP* expression levels were found to be positively correlated with M2 macrophage abundance (Figure 7C).

Therefore, we calculated the correlation of *NREP* with M2 surface markers using the TIMER database and observed a positive correlation between *NREP* expression and *MRC1* (CD206; *R* = 0.34, *P* < 0.001) and *CD163* (*R* = 0.37, *P* < 0.001) expression (Figure 7D). This series of results suggested the presence of a positive association between *NREP* expression and M2 macrophage infiltration. To further investigate the influence of *NREP* overexpression on M2 macrophage abundance in GC, we established a tumor–macrophage cell co-culture model using a transwell non-contact co-culture unit (Figure 7E). We observed that *NREP* overexpression significantly up-regulated the surface markers of M2 TAMs (CD206 and CD163; Figures 7F,G).

Hence, our findings confirmed the positive correlation between *NREP* levels and the abundance of M2 macrophage infiltration.

## Discussion

Gastric cancer is a very common form of cancer (Machlowska et al., 2020). Previous studies have shown that a large number of genetic and epigenomic alterations in oncogenes as well as genetic instability together govern gastric carcinogenesis, a multistep process that involves the interactive regulation of numerous molecular networks (Chia and Tan, 2016). Thus, the search for new oncogenes and biomarkers not only helps in developing new antitumor drugs, but also helps to broaden the known tumor-associated molecular network (Gyurkó et al., 2013). However, gastric tumors contain more than just cancer cells; they are a complex ecosystem composed of several different types of cells and cytokines, all of which greatly influence the proliferation, adhesion, movement, invasion, and metastasis of GC. During tumor formation, tumor cells must adhere closely to the ECM and communicate with other cells to form a stromal microenvironment suitable for proliferation and eventually metastasis (Mierke, 2019; Zeng et al., 2019).

As a potentially useful target gene for tumor therapy, *NREP* can not only directly affect the biological characteristics of tumor cells, but also reshape the TME and influence patient prognosis. The association between *NREP* and the TGF-β1 pathway has been clearly demonstrated (Yue et al., 2014). TGF-β1 is the most effective inductor of EMT and has been found to be up-regulated in a variety of tumors (Chen et al., 2017). *NREP* is thought to stimulate the expression of TGF-β1 by promoting the methylation of the *NREP* promoter and activating the *TGF-*β*1* 5′/3′ UTR (Li et al., 2016). Recent studies have revealed that *NREP* promotes renal fibrosis via the TGF-β1 signaling pathway and that the deletion of *NREP* results in delayed burn wound healing (Stradiot et al., 2018). The process of tumor development has been frequently compared to wound healing owing to several shared molecular and biological processes, including neovascularization, ECM remodeling, and fibrosis (Chang et al., 2004). Therefore, it is reasonable to speculate that *NREP* supports cancer metastasis and the formation of the tumor stromal microenvironment.

It is well known that EMT is a very dynamic process and causes several changes in the cellular phenotype, leading to dramatic cytoskeleton remodeling and the facilitation of cell motility (Pastushenko and Blanpain, 2019). After pooling the genes with *NREP*-linked expression levels, we detected a significant enrichment for EMT-related pathways, including growth factor binding and integrin binding. To further validate this result, we predicted the functions of 10 hub genes using the GSCALite online tool and found that they contribute positively toward the EMT phenotype. Further experiments confirmed that the overexpression of *NREP* significantly up-regulated TGF-β1 and activated the EMT phenotype.

Cancer-associated fibroblasts, important constituents of the tumor stromal microenvironment, play a vital role in tumor–stroma crosstalk, promoting tumor development and progression (Liao et al., 2019). They also secrete oncogenic growth factors, produce ECM, and promote EMT (Pape et al., 2020). TGF-β1-mediated signaling in CAFs has been identified as a pivotal mediator (Porcelli et al., 2019). The TIMER online tool showed that the abundance of CAF infiltration was positively correlated with the expression levels of *NREP*. We also performed single-cell-level analyses and found that *NREP* was predominantly expressed in myofibroblasts, which are a subset of activated fibroblasts characterized by the expression of α-SMA. It has been well-documented that myofibroblasts can be derived from pre-existing stromal fibroblasts and drive tumor progression by establishing TGF-β autocrine signaling in a cell-autonomous manner (Kojima et al., 2010). Considering that bone marrow-derived precursors and bone marrow MSCs are among the multiple origins of CAFs (Borriello et al., 2017), we first co-cultured *NREP*-overexpressing GC cells with MSCs and observed a significant increase in the abundance of CAFs. Further experiments showed that *NREP*-overexpressing GC cells had a stronger ability to recruit CAFs. It has been well-established that CAFs are powerful inducers of EMT activation, and therefore, our findings indicate that *NREP* has a vital function in this process.

An essential step in migration is the remodeling of the cytoskeleton — involving the reorganization and rebuilding of the actin cortical cytoskeleton — which promotes movement (Urra et al., 2018). CAF-derived chemokines influence tumor cell motility by modifying the formin-assembled F-actin cytoskeleton (Zhai et al., 2019). In the present study, GSEA suggested that *NREP* may be involved in cytoskeletal remodeling, and subsequent experiments also confirmed that *NREP* significantly up-regulated F-actin expression levels. Our data demonstrated that *NREP* promotes the activation and chemotaxis of CAFs. Therefore, CAFs may be one of the key factors mediating the promotion of cytoskeletal remodeling by *NREP* in GC cells. Notably, *F*-actin depolymerization and the changes in its cellular distribution, i.e., the transfer of actin filaments from the cytoplasm to the nucleus, are obvious in apoptotic cells (May and Machesky, 2001). The manipulation of F-actin remodeling is used as a therapeutic strategy for inducing apoptosis. Resistance to apoptosis is a well-recognized feature of cancer (Giampazolias and Tait, 2016). Subsequent TUNEL staining showed that the overexpression of *NREP* promoted tumor progression in GC, at least in part, by inhibiting apoptosis.

Normal fibroblasts can acquire a CAF phenotype through communication with cancer cells (Kalluri, 2016). CAFs can be derived from a variety of sources, such as endothelial cells, tumor cells that transdifferentiate into mesenchymal cells via EMT, and bone marrow-MSCs. The recruitment of CAFs from the microenvironment is essential for remodeling the tumor’s ECM and allowing tumor motility and metastasis (Monteran and Erez, 2019). Our findings directly confirm the involvement of *NREP* in this complex process.

During the development of GC, a large number of peripheral blood mononuclear cells are attracted to the tumor mesenchyme and transform into TAMs (Sica et al., 2006). Phenotypic analysis has revealed that in progressive GC, infiltrating TAMs often show the immunosuppressive M2 phenotype and play a key role in promoting tumor EMT (Suarez-Carmona et al., 2017). Recent studies have found that tumor cells undergoing EMT promote the M2 phenotype in macrophages by secreting tumor metabolites, and M2 macrophages in turn secrete a variety of cytokines to promote tumor transformation to the mesenchyme, leading to a vicious cycle (Li et al., 2019). CAFs are also known to enhance TAM recruitment in the TME, creating a positive feedback loop between CAFs and TAMs and the ECM (Cho et al., 2018). Therefore, we first analyzed the relationship between the levels of *NREP* and the infiltration of multiple immune cells based on TCGA-STAD data and found that the level of M2 macrophage infiltration was positively correlated with *NREP* expression; moreover, this correlation showed the highest statistical significance. Similar results were obtained from two other independent GEO datasets. Our co-culture experiments also confirmed the positive effect of *NREP* on the levels of CD163 and CD206 (MRC1), which are surface markers of M2 macrophages. Therefore, our findings indicate that NREP promotes M2 macrophage activation — a process that is considered strongly carcinogenic.

Taken together, our results demonstrate that *NREP* is elevated in GC cells and tissues. High *NREP* levels are associated with some clinicopathological features of GC and a poor patient prognosis. Our results show that *NREP* may act as an important player in the complex gene regulatory machinery driving GC via processes such as EMT activation, CAF activation, actin cytoskeleton remodeling, and M2 macrophage infiltration, ultimately promoting tumor development. However, our study has a few limitations. First, *in vivo* experimental evidence was lacking, and there was insufficient clinical evidence. Therefore, more focused research is needed to elucidate the detailed biological functions and mechanistic roles of *NREP* in GC and to uncover the functional and regulatory niches of this gene.

## Data Availability Statement

The original contributions presented in the study are included in the article/Supplementary Material, further inquiries can be directed to the corresponding author/s.

## Ethics Statement

The studies involving human participants were reviewed and approved by the Ethics Committee of the Jiangsu Province Hospital of Chinese Medicine. The patients/participants provided their written informed consent to participate in this study. The animal study was reviewed and approved by the Ethics Committee of the Jiangsu Province Hospital of Chinese Medicine.

## Author Contributions

J-pL and Y-hZ developed the experimental plan. Y-jL and S-hZ performed all experiments. S-hZ and Y-dH analyzed the data. Y-jL and J-pL wrote the manuscript. All authors have read and approved the final manuscript.

## Conflict of Interest

The authors declare that the research was conducted in the absence of any commercial or financial relationships that could be construed as a potential conflict of interest.

## Publisher’s Note

All claims expressed in this article are solely those of the authors and do not necessarily represent those of their affiliated organizations, or those of the publisher, the editors and the reviewers. Any product that may be evaluated in this article, or claim that may be made by its manufacturer, is not guaranteed or endorsed by the publisher.

## Footnotes

1
https://cistrome.shinyapps.io/timer/

2
https://www.oncomine.org/

3
https://www.proteinatlas.org/

4
https://string-db.org/

## Supplementary Material

Supplementary Figure 1 Prognostic roles of 10 hub genes and relationship with sample types in patients from the TCGA-STAD dataset. **(A–J)** Expression of *FBLN1*, *ELN*, *FN1*, *COL1A2*, *LUM*, *COL1A1*, *COL3A1*, *VCAN*, *BGN*, and *ACTA2* in different sample types (normal vs. tumor) based on the TCGA-STAD dataset. High expression, red; low expression, blue. TPM: Transcripts Per Kilobase Million. **(K–T)** Survival analysis based on *FBLN1*, *ELN*, *FN1*, *COL1A2*, *LUM*, *COL1A1*, *COL3A1*, *VCAN*, *BGN*, and *ACTA2* expression.

Supplementary Figure 2 Validation of cell transfection efficiency.
