# Supplementary figures and images for: Overexpression of NREP Promotes Migration and Invasion in Gastric Cancer Through Facilitating Epithelial-Mesenchymal Transition
Source: Front Cell Dev Biol. 2021 Oct 20;9:746194. doi: 10.3389/fcell.2021.746194 (PMC8565479; doi:10.3389/fcell.2021.746194)

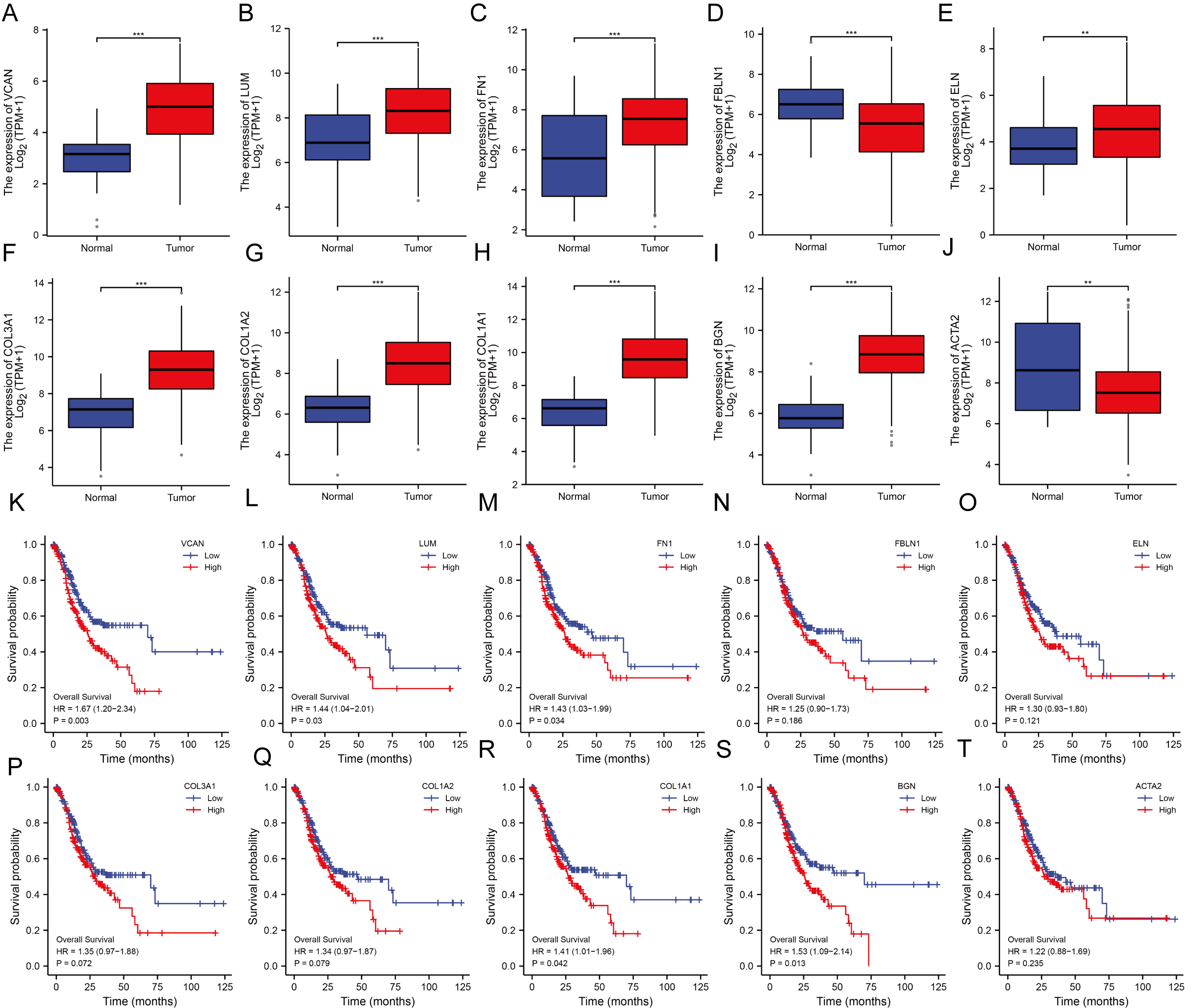

Supplement: Supplementary Figure 1 — Prognostic roles of 10 hub genes and relationship with sample types in patients from the TCGA-STAD dataset. (A–J) Expression of FBLN1, ELN, FN1, COL1A2, LUM, COL1A1, COL3A1, VCAN, BGN, and ACTA2 in different sample types (normal vs. tumor) based on the TCGA-STAD dataset. High expression, red; low expression, blue. TPM: Transcripts Per Kilobase Million. (K–T) Survival analysis based on FBLN1, ELN, FN1, COL1A2, LUM, COL1A1, COL3A1, VCAN, BGN, and ACTA2 expression. [file Image_1.tif]

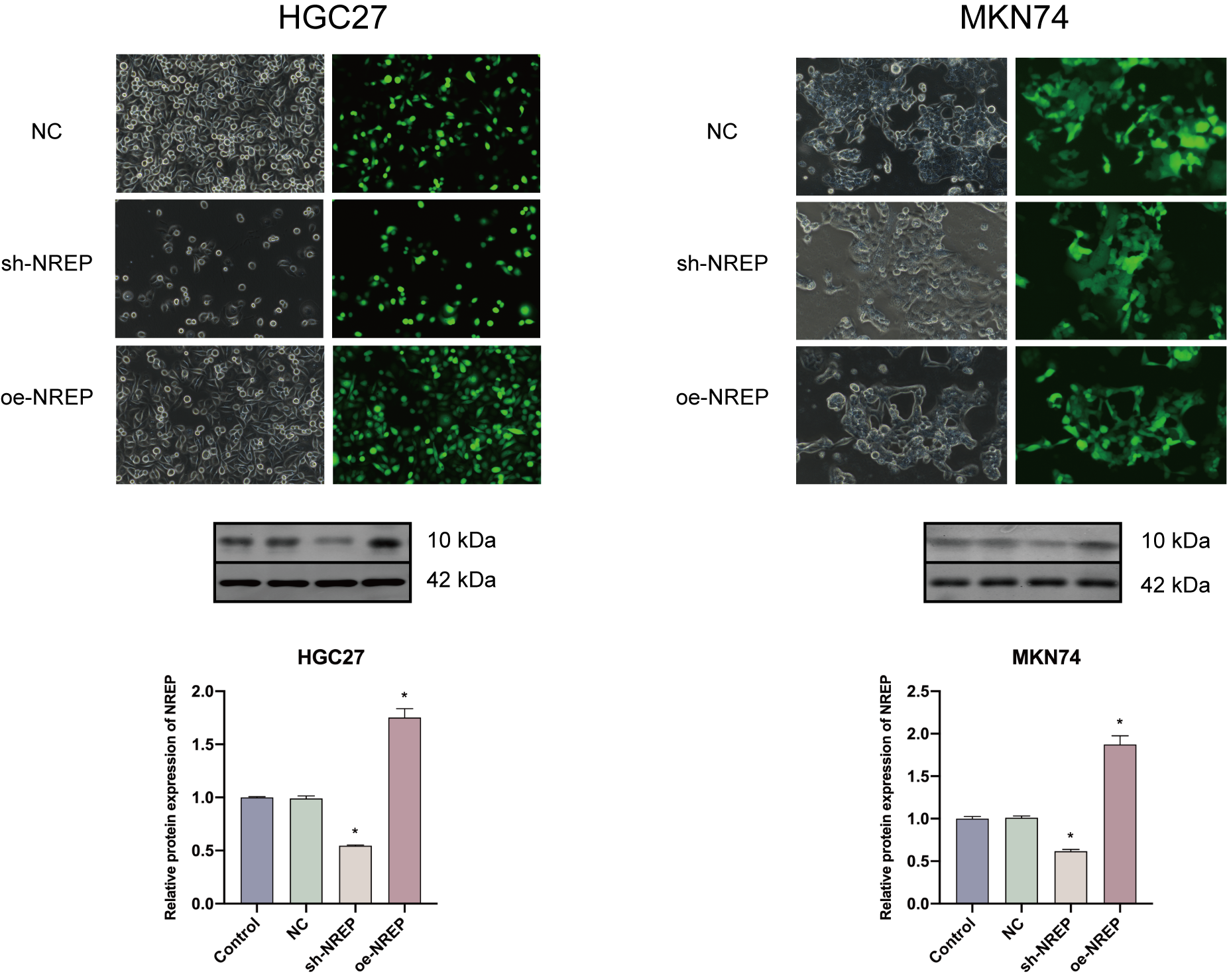

Supplement: Supplementary Figure 2 — Validation of cell transfection efficiency. [file Image_2.tif]
